# Associations Between Amplification (1q) and Prior Cancer in a Real-World De Novo Myeloma Cohort

**DOI:** 10.1093/jncics/pkaa111

**Published:** 2021-01-04

**Authors:** Elizabeth B Lamont, Andrew J Yee, Stuart L Goldberg, David S Siegel, Andrew D Norden

**Affiliations:** 1 COTA, Boston, MA, USA; 2 Massachusetts General Hospital Cancer Center, Boston, MA, USA; 3 John Theurer Cancer Center, Hackensack, NJ, USA

## Abstract

Genomic biomarkers inform treatment in multiple myeloma (MM), making patient clinical data a potential window into MM biology. We evaluated de novo MM patients for associations between specific MM cytogenetic patterns and prior cancer history. Analyzing a MM real-world dataset, we identified a cohort of 1769 patients with fluorescent in situ hybridization cytogenetic testing at diagnosis. Of the patients, 241 (0.14) had histories of prior cancer(s). Amplification of the long arm of chromosome 1 [amp(1q)] varied by prior cancer history (0.31 with prior cancer vs 0.24 without; 2-sided *P* = .02). No other MM translocations, amplifications, or deletions were associated with prior cancers. Amp(1q) and cancer history remained strongly associated in a logistic regression adjusting for patient demographic and disease attributes. The results merit follow-up regarding carcinogenic treatment effects and screening strategies for second malignancies. Broadly, the findings suggest that analyses of patient-level phenotypic-genomic real-world dataset may accelerate cancer research through hypothesis-generating studies.

Increasingly, genomic biomarkers direct clinical care in oncology. Real-world datasets (RWD) from patients treated in usual care settings (rather than in clinical trials) have been studied for nearly 50 years to make inferences about health-care use and outcomes (1). Historically, RWD sources largely comprised preexisting administrative data (eg, billing claims, cancer registry information), which were collected for purposes other than research. RWD in oncology have begun to evolve in the past 5 years, driven by interest in high-quality clinical data for prospective research. Data are now often from electronic health records (EHRs). This new type of RWD, or next-generation RWD (NG-RWD), may contain both phenotypic and genotypic information. Recently, researchers confirmed clinical trial findings with NG-RWD, describing associations between genomic variants (“actionable mutations”) and treatment outcomes in lung cancer patients (2).

In this study, we posited that NG-RWD may be useful in generating hypotheses regarding cancer biology among a specific group of patients with cancer who are often unrepresented on clinical trials: patients with prior cancers.

As patients with newly diagnosed multiple myeloma (MM) routinely undergo fluorescent in situ hybridization (FISH) cytogenetic testing, we studied patients newly diagnosed with MM in a commercial NG-RWD to explore associations between MM-related genomic variants and histories of non-MM cancers. The data were composed of elements abstracted from EHRs from collaborative academic and community-based oncology practices. We identified patients diagnosed with MM between 2010 and 2018, a time frame when FISH characterization was routinely performed (3,4). Among the 2380 MM patients, 1769 (0.74) had FISH testing during the 120 days surrounding their date of diagnosis. Operationalizing “history of cancer” as an EHR recorded non-MM cancer diagnosed any time prior to MM diagnosis through 29 days following MM diagnosis, we compared results of FISH testing [translocation of chromosomes 4 and 14 (t(4; 14)), translocation of chromosomes 6 and 14 (t(6; 14)), translocation of chromosomes 11 and 14 (t(11; 14)); translocation of chromosomes 14 and 16 (t(14; 16)), translocation of chromosomes 14 and 30 (t(14; 20)), deletion of the short arm of chromosome 1(del(1p)), deletion of chromosome 13 (del(13)), deletion of the short arm of chromosome 17(del(17p)), and amplification of the long arm of chromosome 1 (amp(1q))] and histories of prior cancer using χ^2^ tests of proportions. Antecedent non-MM cancer treatment information was not collected. The Western Institutional Review board has reviewed the structure of the dataset and deemed it appropriate for secondary research. All analyses were performed in STATA 14 ML (College Station, TX). All statistical tests were 2-sided, and a *P* value of less than .05 was considered statistically significant.

Table 1 describes the cohort’s demographic and disease attributes. From 1769 patients, there were 263 prior cancers in 241 patients (proportion = 0.14). One prior cancer was noted in 221 of the 241 patients (proportion = 0.92), 2 prior cancers in 19 patients (proportion = 0.08), and 4 prior cancers in 1 patient (proportion < 0.01). Within the cohort, 445 (proportion = 0.25) patients had MM with the amp(1q) FISH-detected genetic variant.

**Table 1.** Description of retrospective cohort (n = 1769)^a^

Variable name	No. (Proportion)	Missing values, No.
Age, median (range)	63 (38-90)	0
Sex		0
Female	771 (0.44)	
Male	998 (0.56)	
Race		82
White	1190 (0.71)	
African American	220 (0.13)	
Asian	40 (0.02)	
Other	237 (0.14)	
Prior cancer		0
No	1528 (0.86)	
Yes	241 (0.14)	
MM immunotype		0
IgG	995 (0.56)	
IgA	355 (0.20)	
IgM	12 (<0.01)	
IgD	5 (<0.01)	
IgE	2 (<0.01)	
Light chain only	387 (0.22)	
Nonsecretory	11 (<0.01	
Minimal secretory	2 (<0.01)	
R-ISS		761
I	253 (0.25)	
II	625 (0.62	
III	130 (0.13)	
FISH +^b^	1769 (1.00)	
amp(1q)	445 (0.25)	1
del(1p)	115 (0.06)	1
del(13)	733 (0.41)	1
del(17p)	339 (0.19)	1
Hyperdiploid	174 (0.10)	1
Hypodiploid	11 (0.06)	1
t(6,14)	5 (<0.01)	1
t(4,14)	173 (0.10)	1
t(11,14)	438 (0.25)	1
t(14,16)	99 (0.06)	1
t(14,20)	17 (<0.01)	1

a The table contains some descriptive attributes of the real-world data cohort pertaining to both patient demographics and disease features as proportions and counts except where indicated. The far right column indicates the numbers of missing observations for the variables described. amp(1q) = amplification of the long arm of chromosome 1; del(1p) = deletion of the short arm of chromosome 1; del(13) = deletion of chromosome 13; del(17p) = deletion of the short arm of chromosome 17; FISH = fluorescent in situ hybridization; MM = multiple myeloma; R-ISS = revised international staging system; t(6,14) = translocation t(6,14); t(4,14) = translocation t(4,14); t(11,14) = translocation t(11,14); t(14,16) = translocation t(14, 16); t(14,20) = translocation t(14,20).

b The full analytic sample (n = 1769) had at least 1 FISH test, although not all patients in the sample had the same FISH tests.

Bivariate analyses showed that the proportion of patients with amp(1q) positivity, a poor prognostic marker in MM, was overrepresented among patients with prior cancers (0.31 with a prior cancer vs 0.24 without; χ^2^ = 5.2, *P* = .02). A nonparametric trend test revealed a strong positive association between the number of patients’ prior cancers (range = 0-4) and amp(1q) positivity (*z* = 2.9, *P* < .01). Antecedent cancers with the highest rates of amp(1q) positivity were prostate cancer, melanoma, lymphoma, and cervical cancer. No other FISH translocations, amplifications, or deletions were associated with prior cancers. Other bivariate analyses showed amp(1q) positivity did not vary by sex but was more frequent among patients 70 years or older at MM diagnosis and among Asian patients compared with Caucasian and African American patients. Additionally, rates of amp(1q) positivity increased with several historically poor prognostic markers in MM.

In a multivariable logistic regression model, there were important associations between amp(1q) positive MM (amp(1q)+MM) (dependent variable) and prior cancers (independent variable) (Table 2). MM patients with 2 or more prior cancers had a 2.79 greater odds (95% confidence interval [CI] = 2.31 to 3.32) of amp(1q)+MM than MM patients without prior cancers after adjusting for patient demographic and disease attributes (and adjusting standard errors for clustering of patients within data collaborators). Patients with a history of prostate cancer had twice the odds of amp(1q)+MM compared with MM patients without that history (odds ratio [OR] = 2.09, 95% CI = 1.94 to 2.19). Amp(1q) was positively associated with other poor MM prognostic factors (ie, advanced revised-international staging system stage, IgA immunophenotype, and kappa light chains). Patients 70 years or older at MM diagnosis had a 25% greater odds of amp(1q) positivity than younger patients (OR = 1.25, 95% CI = 1.17 to 1.34), and Asian patients had more than a 50% increase in odds of amp(1q) positivity compared with Caucasian patients (OR = 1.59, 95% CI = 1.21 to 2.11).

**Table 2.** Adjusted odds of FISH + amp(1q) at diagnosis of multiple myeloma (n = 1768)^a^

Variable name	OR (95% CI)
Age ≥70 y^b^	1.25 (1.27 to 1.34)
Sex	
Female	1.00 (Referent)
Male	1.00 (0.94 to 1.08)
Race	
White	1.00 (Referent)
African American	1.12 (0.87 to 1.46)
Asian^b^	1.59 (1.21 to 2.11)
Other	1.14 (0.77 to 1.70)
Missing^b^	0.67 (0.57 to 0.78)
R-ISS	
I	1.00 (Referent)
II^b^	1.27 (1.01 to 1.60)
III^b^	1.88 (1.24 to 2.85)
Missing	0.75 (0.50 to 1.14)
Light chain type	
Non-lambda light chain	1.00 (Referent)
Lambda light chain^b^	1.31 (1.27 to 1.36)
Immunotype	
Non-IgA	1.00 (Referent)
IgA^b^	1.46 (1.38 to 1.55)
Cancer history	
No other cancer	1.00 (Referent)
1 other cancer	1.10 (0.85 to 1.42)
≥2 other cancers^b^	2.77 (2.31 to 3.32)
No prostate cancer history	1.00 (Referent)
Prostate cancer history^b^	2.06 (1.94 to 2.19)

a Multivariable logistic regression estimating associations between the presence of amp(1q) positivity (dependent variable) and independent variables of substantive interest. amp(1q) = amplification of the long arm of chromosome 1; CI = confidence interval; FISH = fluorescent in situ hybridization; R-ISS = revised international staging system.

b Indicates associations with amp(1q) where 95% confidence interval does not include 1.00. The number of observations for the multivariable logistic regression is 1768 rather than 1769 because 1 member of the cohort (Table 1) had no value for the specific FISH test “amp(1q).”

Using NG-RWD, we found that patients with newly diagnosed MM and histories of other cancers had a higher odds of the poor prognostic risk amp(1q)+MM (5) compared with MM without such histories. Patients with malignancies including prostate cancer and metachronous MM have been described in the literature, although MM FISH findings are not reported (6). Variations in (1q) have also previously been reported among patients with prostate and lymphoid cancers, but to our knowledge, this is the first study to identify an association between amp(1q)+MM (as distinct from other MM subtypes) and a prior history of cancer (7,8). This relationship merits further study. Candidate hypotheses include prior treatment of the initial non-MM malignancy predisposes patients to amp(1q)+MM, a common germline factor predisposes patients to amp(1q)+MM and other cancers, and epigenetic modification confers broad susceptibility to amp(1q)+MM and other cancers (9,10).

Internal validity is a well-known limitation of RWD that can be manifest as imprecise or missing values. In our study of 1769 patients, race was characterized as “other” for 237 (0.13), and the value was missing for another 81 (0.05). The limitation constrains our ability to study the apparent excess in amp(1q) positivity among Asian patients. Challenges such as these underscore the utility of RWD as a scientific adjunct to clinical trial data rather than as a substitute for it.

This study suggests that NG-RWD with patient-level phenotypic-genotypic elements may provide an efficient and comparatively inexpensive tool for exploratory analyses of cancer biology in populations who are underrepresented in clinical trials. We studied patients with dual malignancies (ie, MM and antecedent non-MM cancer[s]), a group commonly excluded from clinical trials. NG-RWD may be leveraged to generate novel hypotheses across a broad scientific scope, ranging from the US population in epidemiology and health-care policy research now to the individual cell in cancer biology research.

## Funding

This was not an externally funded project.

## Notes

**Role of the funder:** Not applicable.

**Author contributions:** EBL—conceptualization, methodology, formal analysis, investigation, resources, data curation, writing – original draft, writing – review and editing, project administration. AJY—methodology, writing – review and editing. SLG—methodology, writing – review and editing. DSS—methodology, writing – review and editing. ADN—resources, data curation, writing – review and editing.

**Disclosures:** EBL: COTA employee at the time of the analyses and creation of first draft (September 20, 2019). AJY: paid consultant for Adaptive Biotechnologies, Amgen, Bristol-Myers Squibb, Celgene, Janssen, Karyopharm, Oncopeptides, Sanofi, and Takeda and receives research funding from Bristol-Myers Squibb and Celgene. SLG: COTA equity. DSS: holds COTA equity. and: COTA employee at the time of the analyses, holds COTA equity.

**Prior presentations:** An early version of this research was presented at the 2019 American Society of Hematology meeting in Orlando, Florida, in December 2019. The published abstract has been included with the submission of this manuscript.

**Acknowledgements**: We are grateful to Dr Ben Tycko for his contribution to this study.

## Data Availability

The data underlying this article were provided by COTA by permission. Data will be shared on request to the corresponding author only with the permission of COTA.
